# Supplementary figures and images for: Nanopore direct RNA sequencing (DRS) of MS2 bacteriophages in E. coli throughout its life cycles reveals a complex transcriptional activity to control and maintain its growth
Source: Virol J. 2026 Mar 6;23:99. doi: 10.1186/s12985-026-03121-5 (PMC13077995; doi:10.1186/s12985-026-03121-5)

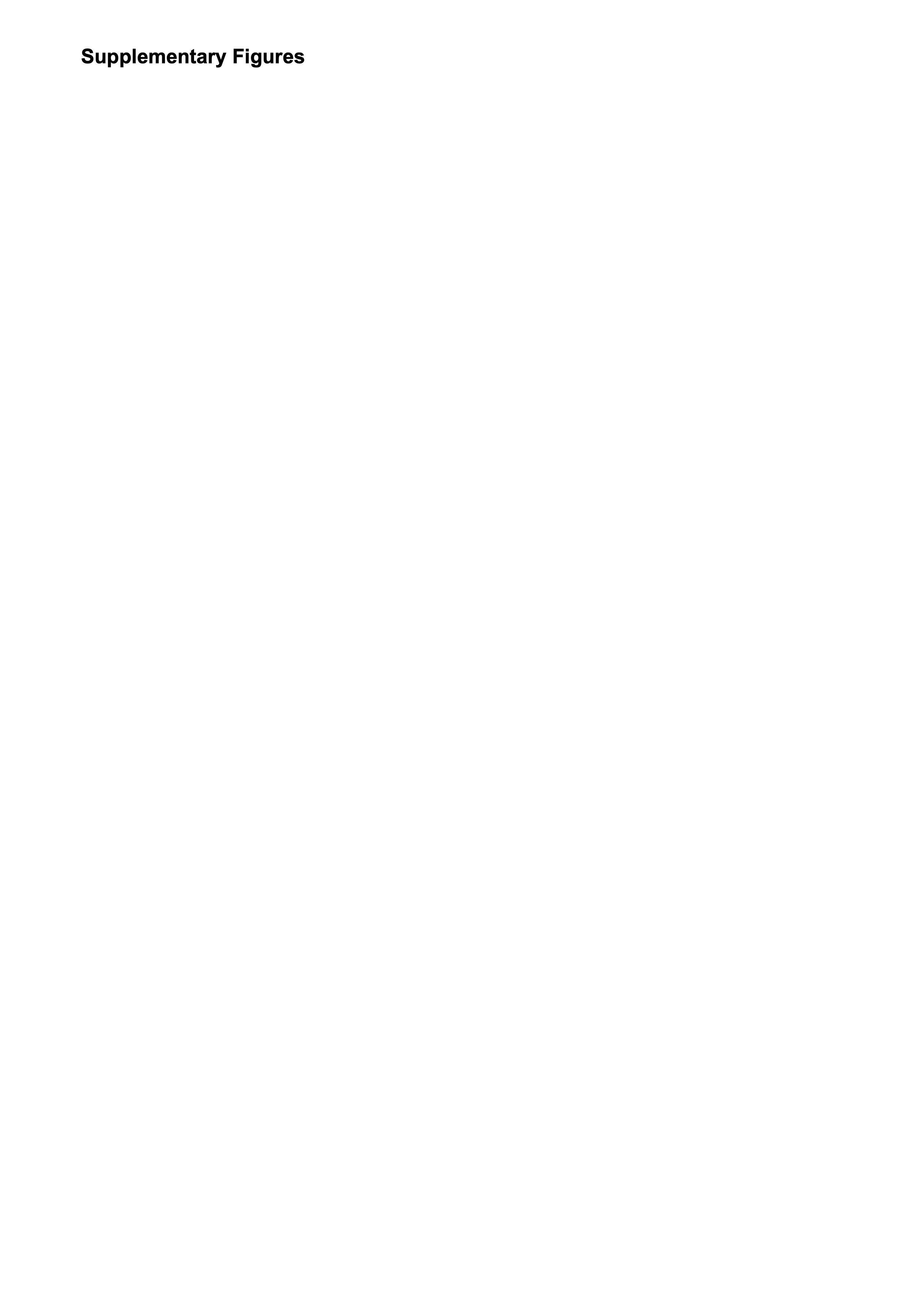

Supplement: Supplementary file 2 — Supplementary Material 2 [file 12985_2026_3121_MOESM2_ESM.png]
